# Clinical outcome of neonates with Carbapenem-resistant *Enterobacteriaceae* infections at the King Edward VIII Hospital’s neonatal unit, Durban, South Africa

**DOI:** 10.4102/sajid.v36i1.223

**Published:** 2021-01-21

**Authors:** Bongani W. Mzimela, Ntombifikile M. Nkwanyana, Radhika Singh

**Affiliations:** 1 Department of Paediatrics and Child Health, College of Health, University of KwaZulu-Natal, Durban, South Africa; 2 Discipline of Public Health Medicine, School of Nursing and Public Health, University of KwaZulu-Natal, Durban, South Africa

**Keywords:** Carbapenem-resistant *Enterobacteriaceace*, multidrug resistance, neonatal sepsis, neonatal mortality, nosocomial infection, prematurity, neonatal outcomes, gram-negative

## Abstract

**Background:**

Carbapenem-resistant infections in neonates are increasing worldwide. These organisms are associated with poor outcomes because of the severity of the disease, lack of treatment options and impaired immune systems of premature neonates. These infections are associated with significantly higher morbidity, mortality and prolonged hospitalisations, especially in developing countries.

**Methods:**

A retrospective study was conducted to evaluate the prevalence and clinical outcomes of neonates with Carbapenem-resistant *Enterobacteriaceae* (CRE) infection over 24 months, from January 2015 to December 2016. All charts for neonates with positive cultures were reviewed, including characteristics of neonates that acquired the infection, possible risk factors and outcomes.

**Results:**

A total of 32 cases were included with a prevalence of 5/1000 admissions. The mortality rate was 0.6/1000, with case facility rate at 12.5%. Most neonates developed CRE infections within the first 7 days of admission. There was an equal distribution between early neonatal deaths (ENND) and late neonatal deaths (LNND). Neonates (34.4%) had prior exposure to Carbapenem, with a higher mortality rate of (75%). There was zero mortality in the HIV-exposed group.

**Conclusion:**

Neonates developed CRE much earlier than previously reported. Invasive procedures on admission carry an associated higher risk for developing CRE, more than the length of stay as previously stipulated. Prevalence of CRE seems to be high in middle-income countries with higher mortality. Thus, strict infection prevention and control (IPC) measures during admission and during the first weeks of life can decrease the incidence and outcome of CRE-related mortality and morbidity.

## Introduction

Over the past decade, there has been a gradual increase in Carbapenem-resistant *Enterobacteriaceae* (CRE) cases reported in neonatal settings. Infections with these multidrug-resistant gram-negative organisms, especially *Enterobacteriaceae*, are of concern in preterm infants and neonates, indicating a break in infection prevention and control (IPC) practices. Neonatal sepsis caused by these pathogens is increasing, and there are limited choices available for treatment.^1^ Carbapenem-resistant organisms were already described as a cause of neonatal septicaemia in India as early as in 2007.^2^ The Centers for Disease Control (CDC) reported an increase in CRE cases from 0.04% in 1998 to 3.8% in 2008 and estimated cases to be at 9.7% in 2018.^3^ The latest reports already report the prevalence to be around 6.7% worldwide, although this might just be the reflection of an improvement in microbiological diagnosis and screening techniques.^1^ The high mortality rate associated with CRE infections, especially in adults, makes the rise in prevalence a worrying factor. It is, therefore, no surprise that much attention is now being placed on disease prevention. However, minimal information is known about disease mortality in neonates.

Carbapenem-resistant *Enterobacteriaceae* infections are known to be associated with significant morbidity and mortality, and these pathogens are now reported to be on the increase in children and neonates.^4^ Current reports indicate that CRE infections cause severe and even life-threatening infections in the most vulnerable populations.^5,6,7^ Since the first outbreak of CRE reported in 2003, the incidence of CRE infections has increased rapidly and emerged in over 36 countries worldwide.^5,7,8^

The risk factors for CRE infection and colonisation have been well described in the adult literature. However, the description of CRE in paediatric patients has been limited to a few case reports.^5^ A study done by Dirajlal-Fargo et al. reported that very few cases of CRE had complications or showed detrimental outcomes as only one out of 13 children progressed to having a systemic infection. Prolonged hospital stays proved to be the most important and independent risk factor for developing CRE.^3^

Early recognition of CRE colonisation is essential for timely implementation of control measures to reduce patient-to-patient transmission, outbreaks and infection-related morbidity and mortality, which is said to be 28% higher in CRE neonates than controls.^9,10^ However, one of the previous outbreak investigations identified asymptomatically colonised patients, as well as mechanical ventilators and hospital plumbing, as reservoirs of CRE that facilitate nosocomial spread despite rigorous infection control procedures.^7^

A retrospective chart review was conducted to delineate the prevalence and incidence of CRE infections and to also look at outcomes of neonates with CRE infection at King Edward VIII Hospital’s neonatal unit, a tertiary hospital in Durban, KwaZulu-Natal (KZN). This study was conducted to establish mortality and morbidity of CRE in a neonatal population over a 2-year study period. Attempts to establish patterns and characteristics of the neonates and their risk factors were made.

## Materials and methods

A quantitative, retrospective, descriptive observational study was conducted at King Edward VIII Hospital, a tertiary hospital in KZN, with a 40 bedded nursery which includes four ICU and eight high care beds, over a 2-year study period between 01 January 2015 and 31 December 2016. All the neonates with CRE infections confirmed by the Microbiology Laboratory were included in the study and neonates transferred in from other hospitals were excluded. Rectal swabs, blood culture, full blood count and C-reactive proteins were done on admission for all neonates as part of the septic workup. Subsequently repeat cultures were done if a neonate had features suggestive of hospital-acquired infection. These cultures included blood culture, urine culture, cerebrospinal fluid (CSF) analysis and endotracheal aspirates (if the neonate was ventilated).

According to the unit protocol, the first-line antibiotics used in the unit for suspected sepsis were Ampicillin and Gentamycin. Second-line antibiotics for suspected hospital-acquired infection were Piperacillin-Tazobactam and Amikacin. Third-line antibiotics were Carbapenems; predominately in our unit we were using Meropenem as third line and antibiotics were changed based on culture and sensitivity of the organism once results are available.

Once advised of a positive culture by the Microbiologist, the patient details were recorded in the culture book as part of the surveillance.

Files of the neonates with a positive culture of CRE were retrieved from medical records.

## Case definitions

*Carbapenem-resistant Enterobacteriaceae infection* was diagnosed when CRE was isolated from any given body site that was associated with clinical manifestations of infection. Hospital-acquired infection was defined as CRE infection detected ≥ 48 h after hospital admission and not incubating at the time of hospitalisation.

*Carbapenem-resistant Enterobacteriaceae* was defined or confirmed when the organism identified showed resistance to one or more of the Carbapenem antibiotics using the Vitex (bioMerieuxSA, France) automated system with minimum inhibitory concentration (MIC) > 2 was used and thereafter a confirmation was done with Epsilometer test (E test) also showing MIC > 2 for Carbapenem.

*Neonatal sepsis* refers to a clinical syndrome consisting of nonspecific symptoms and signs of infection accompanied by bacteraemia in the first 28 days of life.

*Early neonatal death (ENND)* was defined as the death of a newborn between 0 and 7 days of life.

*Late neonatal death (LNND)* was defined as the death of a newborn after 7 days of life.

## Data collection

Clinical information of neonates confirmed to have an infection caused by CRE (including age, gender, clinical manifestations at the time of the CRE culture result, HIV status, and antibiotic used preceding CRE infection, antibiotic treatment of the CRE infection and outcome of neonates) was extracted from the hospital records. Charts were analysed to collect other relevant data which included antenatal history, risk factors (which included invasive procedures, duration of stay, ventilation, gestational age) and outcomes.

Microbiological information on the CRE isolates cultured from children infected or colonised with CRE was extracted from the National Health Laboratory Services (NHLS) microbiology database, including genus and species, results of selective Carbapenemase antibiotic sensitivity pattern. All data were entered on standardised data-sheets. The cultures were confirmed using the standard laboratory services, and specimens were collected using standard sterile methods.

## Data analysis

Data were initially captured anonymously into an Excel 2010 spreadsheet (Microsoft, USA) and analysed using descriptive statistical methods (SSPS version 23). Demographics, clinical, laboratory features and results were presented as mean and standard deviations for quantitative variables (age, birth weight, duration of stay) and as frequencies and percentages for qualitative variables (gender, gestational age at birth, admitting diagnosis, morbidity and mortality).

### Ethical consideration

Ethical approval for the study was obtained through the University Biomedical Research Ethics Committee (BREC: BE337/16). Hospital approval was obtained through the department of health. No consent was required from participants as this was retrospective chart review.

## Results

Over the 2-year study period, 36 neonates with CRE infection were identified from 6443 admissions. Four did not meet the criteria and were excluded. Of these three patients who came with CRE infection from other institutions, one was admitted to the paediatrics ward.

Thirty-two (*n* = 32) patients were included in the study with an infection prevalence rate of 5/1000 admissions. The average gestational age was 34 weeks (range 28–42 weeks), with an average birth weight of 1600 grams (g) (range 850 g – 4240 g). Fifty-six point three per cent (56.3%) (*n* = 18) of patients were male, 65.8% (*n* = 21) of the patients had birth weight below 2.5 kilograms (kg), and the very low birth weight (VLBW) category between 1500 g and 2500 g had the highest mortality of 50%. Baseline characteristics of the neonates are shown in Table 1. The parity of the mothers was non-contributing as the majority of the mothers were either para1 (37.5%) or para 2 (34.4%).

**TABLE 1:** Characteristics and demographic for Carbapenem-resistant *Enterobacteriaceae*-infected neonate.

Characteristic	*n* (32)	%	Mortality (*n* = 4)	Mortality per variable (%)
**Gender**				
Male	18	56.3	0	0.0
Female	14	43.8	4	28.6
**Birth weight**	-	-	-	-
< 1000 g	8	25.0	1	12.5
1000 g – 1499 g	7	21.8	0	0.0
1500 g – 2499 g	6	18.9	2	33.3
> 2500 g	11	34.4	1	9.0
Average gestational age	34 weeks	-	-	-
**Maternal factors**				
Virginal delivery	10	31.2	2	18.2
C/S	22	68.8	2	9.5
HIV exposed	11	34.4	0	0.0
RVD unexposed	21	65.6	4	19.0
Syphilis exposed	0	0.0	0	0.0
Booked	31	96.9	3	9.7
Unbooked	1	3.1	1	100
**Unit risk factor**	-	-	-	-
TPN	13	40.6	2	15.4
UVC line	25	78.1	4	16.0
Vent support	25	78.1	4	16.0
Carbapenem exposed	11	34.4	3	27.2
Carbapenem unexposed	21	65.6	1	4.8

Note: Average days: TPN = 7; UVC line = 6.5; Vent support = 4.

CRE, Carbapenem-resistant *Enterobacteriaceae*; C/S, cesarean section; RVD, retroviral diseases; TPN, total parenteral nutrition; UVC, umbilical vein catheterisation.

Newborn’s by Caesarean section was 68.8% (*n* = 22). The most common indications for a Caesarean section were preterm labour, gestational hypertension with associated complication and foetal distress. The remaining deliveries, 31.2% (*n* = 10), were vaginal delivery. There were no assisted deliveries.

Neonates (31.3%, *n* = 10) required resuscitation at birth, with low Apgar’s scores corresponding with the need to resuscitate.

## Results on risk factors

The average number of days spent in the unit was 2.5 days on non-invasive respiratory support and average for intermittent positive pressure ventilation (IPPV) was 2 days. The average duration of the umbilical venous catheter (UVC) and umbilical arterial catheter (UAC) lines was 6.5 and 2 days, respectively. Umbilical venous catheter and respiratory support had an equal contribution to mortality (Table 1). Total parenteral nutrition (TPN) duration was an average of 4 days. The peripherally inserted central catheter (PICC) averaged 4 days. The presence of UAC lines did not contribute to the risk of infection, and three of the patients who demised were exposed to them.

The majority (*n* = 27) of patients were exposed to Ampicillin, Gentamycin, Amikacin and Piperacillin-Tazobactam on an average of 4 days each prior to CRE diagnosis. Only 34.4% (*n* = 11) had prior exposure to Carbapenems before CRE positive culture, but three (75%) of the four patients who demised had prior exposure to Carbapenems before the culture date on days 1, 4 and 7, respectively. There was high mortality in patients who had prior exposure to Carbapenems (3/11) 27% compared to the unexposed group (1/21) 5%.

Indication for admission was non-contributory as risk factors, and the trends were consistent with general indications for admissions in the neonatal unit.

**FIGURE 1:**
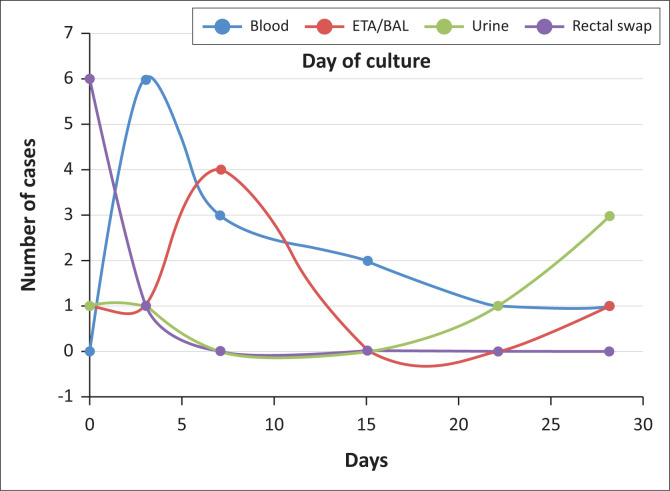
Comparing date to positive culture for different specimens type.

The average stay in the nursery before a positive CRE culture was 7 days, with a range from 1 to 59 days. The average day of life was 10.5 days (Figure 1).

**FIGURE 2:**
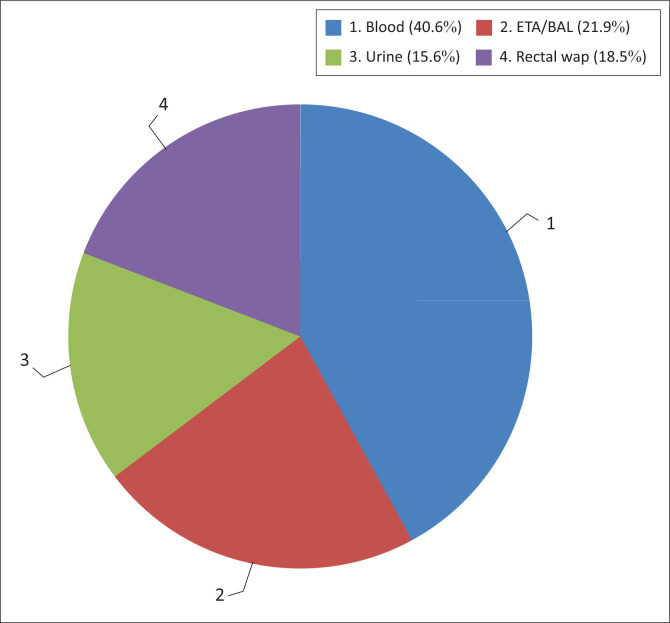
Distribution of culture results according to specimen type.

The most common site of culture was blood *n* = 12 (37.5%), followed by endotracheal aspirates *n* = 7 (21.9%), CSF at *n* = 1 (3.1%) and lastly pus swab at *n* = 1 (3.1%) (Figure 2). The pus swab was from one patient who had drip site ulcers on the left forearm. Rectal swabs done on admission to rule out colonisation were positive in *n* = 6 (18.8%) of the neonates and only *n* = 5 (15.6%) had positive urine culture results. A positive urine culture was on average around day 24 of life, in keeping with possible colonisation or late-onset sepsis.

## Organisms and sensitivity

The most common organism was *Klebsiellapneumoniae* 20/32 (62.5%), followed by *Escherichia coli*8/32 (25%), *Enterobacter cloacae*, 3/32 (9.4%) and *Citrobacterfreundii* 1/32 (3.1%); 25/32 (78.1%) organisms were sensitive only to Colistin (polymyxin E), with 4/32 (12.5%) cultures, (two on blood and two on urine), resistant to everything including Colistin. These four patients were treated with Meropenem infusion plus Colistin, and all four survived. The remaining 3/32 (9.4%) organisms were sensitive to Amikacin, Tigecycline and Ciprofloxacin, respectively.

## Blood parameters

Haematological changes that were noted were severe thrombocytopenia (platelets less than 50 cells/m^3^) on the day of positive culture, with severe neutropenia compared to the admission results (Table 2). However, there was equal distribution in the ranges of WCC, and 50% distribution between normal platelet counts versus the abnormal platelet count on the day of positive culture.

**TABLE 2:** Haematological finding on admission and day of culture.

Haematological Findings on admission versus on the day of culture-positive					

Mortality		On admission		On the day of culture	
*n*	%	*n*	%	*n*	%
**Platelets**					
> 150					
0	0.0	23	71.9	16	50
100–149					
1	20.0	6	18.8	5	15.6
99–50					
1	25.0	3	9.4	4	12.5
< 50					
2	28.6	0	-	7	21.9
**White cell count**					
> 20					
1	16.7	11	34.4	6	18.8
5–19.9					
2	10.0	21	65.6	20	62.5
< 5					
1	16.7	0	-	6	18.8
**C-reactive protein**					
< 5					
0	0.0	31	96.9	13	44.9
5–100					
1	20.0	1	3.1	5	17.2
> 100					
3	27.3	0	0.0	11	37.9

None of the positive rectal swabs (6/32) were treated. Two of the urine results were not treated as the patients were clinically stable at the time of getting results. One patient demised before we had results of *E. coli*, which was sensitive to Colistin. The remaining 23/32 (71.9%) patients were treated according to sensitivity. For the Colistin sensitive patients, four were treated with Colistinmonotherapy, and in the others, Colistin was used in combination with Meropenem, and as stated earlier, the Meropenem was changed to a continuous infusion in those patients with Colistin resistance. The three remaining patients who died had organisms that were sensitive to Colistin, one died on day 3 of Colistinand Meropenemand the other two died on day 7 of Colistinand Meropenem.

## Mortality

Overall mortality was 12.5% (*n* = 4/32), with an equal (50/50) distribution between early (*n* = 2) and late (*n* = 2) neonatal deaths (NNDs) (Table 3). The two early NNDs were because of severe hypoxic ischemic encephalopathy (HIE), and the others were because of overwhelming sepsis. From the late NNDs, one was an 860 g, who did well initially but developed sepsis on day 20 of life and a second baby was a cardiac patient who developed sepsis 1-week post-cardiac repair on day 35 in hospital. None of the four patients who demised were HIV exposed.

**TABLE 3:** Outcome of *Carbapenem-resistant Enterobacteriaceae*-infected neonates.

Outcome – mortality	*n* (32)	*N*	%
Discharged	28	-	87.5
Demised	4	-	12.5
ENND	2	-	6.25
LNND	2	-	6.25
**Mobility of discharge**	*N* (28)	-	-
BPD/CLD	7	-	21.9
HIE	7	-	21.9
ROP	4	-	12.5
PHH	3	-	9.4
None	7	-	21.9

CRE, Carbapenem-resistant *Enterobacteriaceae*; ENND, early neonatal death; LNND, late neonatal death; BPD/CLD, bronchiopulmonary dysplasia/chronic lung disease; HIE, hypoxic ischemic encephalopathy; ROP, retinopathy of prematurity; PHH, post hemorrhagic hydrocephalus.

*Klebsiellapneumoniae* accounted for 50% of the mortality, while *E. coli* and *C. freundii* contributed 25%, respectively.

## Discussion

The finding in this study showed a prevalence of CRE similar to that seen in middle-income countries.^2,9,11,12^ However, it is higher than that seen in developed countries.^13^

Mortality was below that reported in the literature.^10^ This was partly because of the poor yield of the cultures and the small number of patients. Most of the patients did not have prior exposure to Carbapenem, which is in keeping with current trends noted around the world that patients are developing CRE without exposure to any of the Carbapenem.^14^ However, patients who received Carbapenem prior to developing CRE on cultures showed higher mortality than the unexposed group, which was in contrast to the finding by Ballot.^9^ This could be attributed to the patients been more ill, thus requiring the third-line empirical treatment prior to positive CRE culture.^14^ There was an early yield of a positive culture for both blood and ETA culture, on an average of 7 days, which was a week earlier compared to other middle-income countries.^9^

One important finding was a positive correlation of full blood count values with the severity of infection and poor outcome. High CRP was associated with high mortality as *n* = 3 (75%) of the patients who demised had a high CRP of above 100 on the day of positive culture.

This study demonstrated that ventilation, respiratory support and umbilical line insertion are possible risk factors for CRE infection. This is in keeping with recent findings of the ventilator and hospital plumbing being a possible reservoir for CRE infections.^7^ Our patients had CRE a week earlier than other local studies.^9^ We could not find any antenatal factors that contributed to CRE infection and our study population was, however, predominantly of low birth weight; the true significance of these as risk factors could not be attained; and this speaks to a local high reservoir with evolving epidemiology for CRE worldwide.^5,3,14^

The recommendation is that the use of septic markers in deciding whether to treat or not should be used in conjunction with antibiotics stewardship and sound clinical judgement. We recommend that CRE infection (if the surveillance indicates a high incidence of CRE in the unit) be considered early in a neonate with sepsis not responding to initial antibiotics after the first week of life in the nursery.

## Conclusion

Neonates developed CRE sepsis much earlier, within the first week of life, than previously reported in other studies. However, mortality is variable and requires further studies. Prevalence of CRE seems to be high in middle-income countries. Stricter IPC measures during admission and the first weeks of life can decrease the incidence and outcome of CRE-related mortality and morbidity.

## Limitations

Challenges with this study were that this was a retrospective study, and the sample size was too small. Our study only used Vitek results to categorise CRE with no MIC values for comparison, no genus species level data and no Carbapenemase-producing *Enterobacteriaceae* (CPE) confirmation test. Long-term follow-up of neonates discharged from the neonatal unit was not evaluated. We recommend that further prospective and multicentre studies are required to delineate outcomes and risk factors for acquiring CRE sepsis.
